# Prenatal Physical Activity, Pre-Pregnancy BMI, and Their Relationship with Gestational Diabetes: A Retrospective-Prospective Single-Center Study

**DOI:** 10.3390/nu17050786

**Published:** 2025-02-24

**Authors:** Martyna Kiljan, Anna Weronika Szablewska

**Affiliations:** Department of Obstetric and Gynaecological Nursing, Institute of Nursing and Midwifery, Medical University of Gdansk, Sklodowskiej-Curie 3 A, 80-210 Gdansk, Poland; kiljan_martyna@gumed.edu.pl

**Keywords:** gestational diabetes, physical activity, prenatal, pregnancy, body mass index

## Abstract

Background: In recent years, there has been an increase in the incidence of gestational diabetes (GDM) with serious risks for both mother and child. Pre-pregnancy BMI and physical activity significantly influence GDM development. Promoting a healthy lifestyle is essential to prevent GDM and improve health outcomes for mother and baby. Objective: The aim of this study was to evaluate the relationship between pre-pregnancy physical activity and pre-pregnancy BMI and the occurrence of gestational diabetes, as well as to assess their impact on the development of complications associated with gestational diabetes. Methods: A retrospective-prospective study was conducted from October 2024 to December 2024 at a tertiary referral hospital in Poland. The study included 205 pregnant women (42 with gestational diabetes, 163 without gestational diabetes) who met the inclusion criteria. Data were collected using a self-administered questionnaire and the Polish version of the Get Active Questionnaire for Pregnancy (GAQ-P). The impact of pre-pregnancy physical activity and pre-pregnancy BMI on the occurrence of gestational diabetes was assessed based on data collected from both surveys and medical records analysis. Statistical analyses included Pearson’s chi-square tests, logistic regression, and Cramér’s V coefficient to determine the relationship between pre-pregnancy physical activity and ppBMI and the occurrence of gestational diabetes. Results: The study revealed that pre-pregnancy BMI and gestational weight gain were significant predictors, with a higher BMI increasing the risk of gestational diabetes. In contrast, physical activity before pregnancy—including its frequency, intensity, and duration—was not a significant predictor. Additionally, no significant associations were found between physical activity and birth weight, mode of delivery, or preterm birth. These findings highlight the role of pre-pregnancy BMI in gestational diabetes risk while suggesting that physical activity before conception may have a limited impact. Conclusions: These results highlight the significant role of pre-pregnancy body mass index (BMI) in the development of gestational diabetes, emphasizing the need for targeted interventions aimed at maintaining a healthy weight before conception. They suggest that weight management strategies should be an important component of gestational diabetes prevention. Future research should further investigate the complex interaction between lifestyle factors and metabolic health to refine preventive recommendations and improve health outcomes for mothers and newborns.

## 1. Introduction

Gestational diabetes mellitus (GDM) is one of the most frequently diagnosed metabolic disorders during pregnancy, posing significant risks of complications for both the mother and the developing fetus [1,2]. This condition, characterized by disturbances in glucose metabolism that first appear during pregnancy, has become a growing global health concern [1]. Although these disturbances often resolve after childbirth, GDM carries long-term health consequences, such as an increased risk of type 2 diabetes in the mother and a predisposition to obesity and metabolic disorders in the child [1,3,4,5].

Moreover, untreated or poorly controlled GDM can lead to various complications, including excessive fetal growth (macrosomia), preterm birth, pregnancy-induced hypertension, and complications during delivery [2,3,4,6,7,8]. These complications not only endanger maternal and fetal health but also contribute to significant healthcare costs. Managing GDM and its associated complications often requires frequent medical monitoring, specialized care during delivery, and neonatal intensive care, all of which place a substantial financial burden on healthcare systems [9,10,11]. Studies have shown that the economic burden of GDM includes increased rates of hospitalization, neonatal intensive care costs, and follow-up treatments for both mothers and children [9,10,12].

The current rise in the number of GDM cases places it at the forefront of public health concerns [1,13]. In Poland, there is also a noticeable increase in the number of pregnant women with diabetes. In 2021, this number exceeded 50,000 annually, accounting for 12.5% of total births, and in 2022, it increased to 16% [14]. The causes of this phenomenon are multifactorial, involving both genetic predispositions and environmental factors, such as lifestyle, diet, and physical activity levels [15,16,17]. Notably, a lack of physical activity and excessive pre-pregnancy body weight have been identified as risk factors for GDM development [16,17,18]. Beyond the immediate pregnancy-related complications, GDM carries significant long-term health consequences. Women with a history of GDM face a much higher risk of developing type 2 diabetes and cardiovascular diseases later in life, while their offspring are more likely to develop obesity and metabolic disorders in childhood and adulthood [3,4,19]. These persistent health challenges highlight the urgent need for effective prevention, early detection, and comprehensive management strategies to mitigate the clinical and economic impacts of GDM [20].

Recent studies suggest that engaging in regular physical activity before pregnancy and maintaining a healthy body mass index (BMI) can significantly reduce the risk of developing this condition [16,17,21]. Physical exercise improves glucose metabolism, enhances insulin sensitivity, and supports the maintenance of a healthy body weight, which are critical for preventing GDM and mitigating its progression [22].

Given the increasing prevalence of high-risk pregnancies, understanding the role of physical activity and BMI in the prevention and pathogenesis of GDM is particularly important [23]. A deliberate approach to preparing the body for pregnancy, incorporating a healthy diet and regular physical activity, can significantly impact the health of both the mother and child during pregnancy and later in life [24].

Recent research suggests a potential association between anemia and gestational diabetes mellitus (GDM). Iron deficiency anemia, in particular, appears to influence glucose metabolism and may contribute to an increased risk of insulin resistance. Conversely, pregnant women diagnosed with GDM may be more susceptible to anemia due to increased nutritional demands and metabolic complications [25].

Considering the multifactorial etiology of GDM, it is crucial to examine the combined impact of pre-pregnancy BMI and physical activity, as both factors play interconnected roles in metabolic regulation. Excessive pre-pregnancy BMI has been strongly linked to insulin resistance, chronic low-grade inflammation, and impaired glucose metabolism, which are key contributors to GDM development [26]. Meanwhile, regular physical activity enhances insulin sensitivity, reduces systemic inflammation, and supports energy balance, mitigating some of the adverse effects of excess body weight. Investigating these factors together allows for a more comprehensive understanding of their interaction and potential for early intervention [15,27]. Addressing both weight management and structured physical activity before conception could serve as an effective strategy in reducing GDM incidence and improving pregnancy outcomes.

The objective of this study was to determine the relationship between pre-pregnancy physical activity, pre-pregnancy BMI, and the risk of developing gestational diabetes, as well as the occurrence of its selected complications. Based on existing evidence, we hypothesized that pre-pregnancy physical activity would serve as a protective factor against GDM by improving insulin sensitivity and metabolic regulation, while a higher pre-pregnancy BMI would be associated with an increased risk of GDM development. Although numerous studies focus on the risk factors for developing gestational diabetes, there remains a lack of research emphasizing innovative preventive strategies, such as the role of preconception care, the optimal type and level of physical activity with protective effects, or the impact of nutritional status prior to pregnancy [28]. Understanding these aspects could introduce a new dimension to GDM prevention, paving the way for more effective and targeted interventions. The findings presented in this study can serve as a basis for developing effective health strategies, educational programs, and guidelines for women planning pregnancy, ultimately contributing to improved overall health outcomes for mothers and children.

## 2. Materials and Methods

### 2.1. Study Design

The present study was a single-center, cohort study conducted in a group of 205 Polish women (in Poland, ethnic minorities make up a relatively small proportion of the total population, so we did not distinguish between ethnic groups) during pregnancy and then after giving birth. We followed the STROBE guidelines for cohort studies [29]. All procedures were carried out in accordance with the principles set out in the World Medical Association (WMA) Declaration of Helsinki for research involving human subjects and were approved by the Bioethics Committee of the Medical University of Gdansk, No. NKBBN/406-1/2024.

This study employed both retrospective and prospective data collection methods. Retrospective data included the occurrence of gestational diabetes mellitus (GDM), as participants had already undergone an oral glucose tolerance test (OGTT) at the time of the study. Additionally, retrospective data covered pre-pregnancy body mass index (ppBMI), coexisting medical conditions, obstetric history, and physical activity levels during the six months preceding conception. These data were gathered through a self-administered questionnaire. Prospective data collection focused on pregnancy outcomes, delivery details, and neonatal health indicators. These outcomes were analyzed during the second phase of the study based on hospital medical records from participants who gave birth at the study site.

A total of 230 surveys were distributed to pregnant women who met the inclusion criteria for the study. Of these, 213 surveys were returned. After excluding 8 surveys due to clinical situations where the pregnancy could have ended in preterm labor, potentially leading to lower maternal weight gain and consequently poorer neonatal outcomes, which could affect the overall interpretation of the results, 205 correctly completed surveys were included in the final analysis.

Women attending the antenatal clinic of a tertiary referral hospital participated in the study. They were invited to join at the start of the third trimester (after the 28th week of pregnancy). This stage was chosen to allow for a detailed assessment of the obstetric condition, considering possible contraindications to physical activity, such as cervical shortening, placental status, and placental location. Additionally, by this point in pregnancy, the participants had already undergone the oral glucose tolerance test, enabling the evaluation of gestational diabetes.

**Figure 1:**
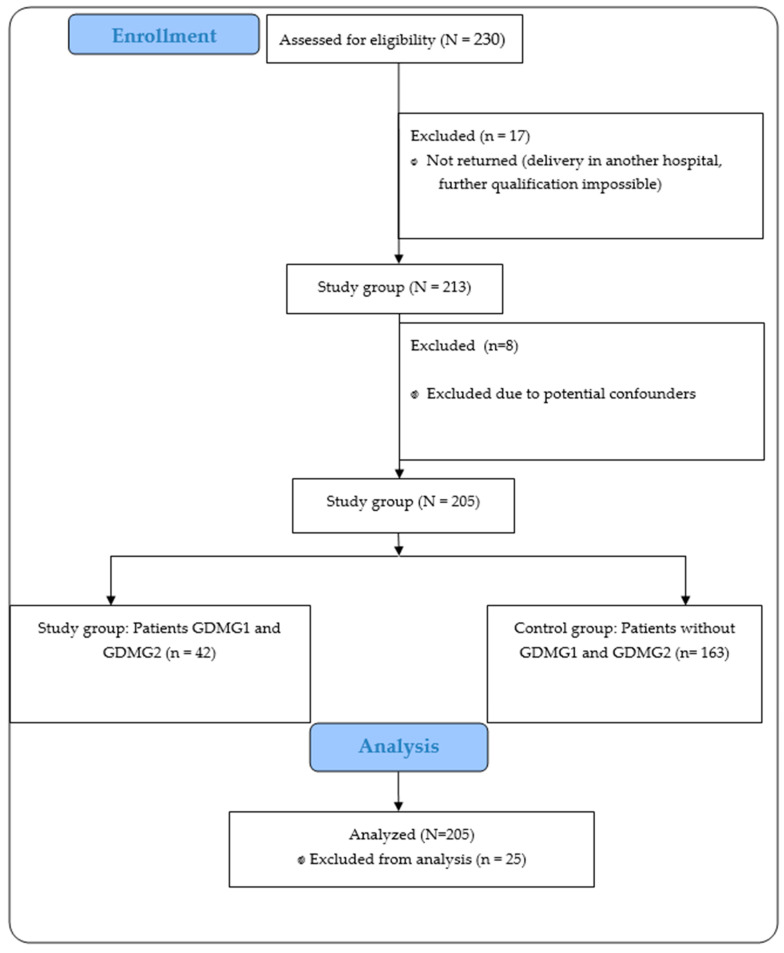
The flow of participants through the study.

In the first stage of the study, the pregnant women completed a questionnaire that collected socio-demographic data, information about their health status, pre-pregnancy BMI (ppBMI), and any contraindications to physical activity. Furthermore, each respondent provided consent to grant access to her and her child’s medical records—if she gave birth at the hospital where the study was conducted—in order to compare the obtained results with delivery and neonatal outcomes. The participants’ flow through the study is presented in Figure 1.

### 2.2. Setting

The study was conducted in one tertiary (level III) referral hospital in the northern region of Poland. The hospital classification system in Poland defines different levels of maternal care: level I focuses on managing physiologically normal pregnancies, childbirth, the postpartum period, and care for healthy newborns with possible short-term pregnancy complications; level II provides for pregnancies with moderate complications; and level III is designated for the most complex and high-risk pregnancy cases. The Polish Society of Gynecologists and Obstetricians recommends that patients diagnosed with gestational diabetes requiring insulin therapy (GDM2) should deliver in a level III referral hospital to ensure optimal medical care for both the mother and the newborn [30]. The period of data collection and patient eligibility was from September 2024 to December 2024.

### 2.3. Participants

All participants were informed about the study’s objectives and gave their voluntary written informed consent to participate by indicating their agreement within the surveys. The principal investigator conducted a personal interview with each woman, clarifying any doubts about the study and the recruitment process. The pregnant women were able to ask questions.

The study included 205 pregnant women (42 women with gestational diabetes mellitus and 163 women without gestational diabetes mellitus) who met the inclusion criteria.

The study focused on patients with gestational diabetes mellitus (GDM), but for comparative purposes, patients without GDM were also included.

The sample size was determined based on the availability of patients meeting the inclusion criteria within the specified time and location of the study. Preliminary data from the Central Statistical Office (pol. GUS, Główny Urząd Statystyczny), as of 23 December 2024, indicate that approximately 214,000 live births were registered between January and October 2024, with 12,600 births occurring in the first half of the year [31]. The prevalence of gestational diabetes mellitus (GDM) in the general population varies between 3–5% [32] and 12–16% [14] depending on the sources and diagnostic criteria used.

Therefore, the inclusion of 42 patients with GDM in our study constitutes a representative sample in the context of local conditions and patient availability, as calculated by the Raosoft program [33]. Additionally, the control group, consisting of 163 patients, was selected in a proportion that allowed for statistical analyses while maintaining a comparable demographic and clinical structure.

### 2.4. Inclusion and Exclusion Criteria

The inclusion criteria encompassed pregnant women aged 18 years or older whose pregnancy was either complicated by gestational diabetes mellitus or without such complications. A self-reported screening tool was used in the study, which allowed for the exclusion of women based on health reasons that may have been potential confounding factors (e.g., due to multiple pregnancies or placenta previa) and/or health conditions that restricted physical activity prior to pregnancy (e.g., due to musculoskeletal disorders, disabilities, or other risks). The screening tool is described in the following section.

### 2.5. Data Collection Tools

Data were collected using a diagnostic survey method. The primary data collection tool was a self-administered questionnaire consisting of two parts.

The first part of the questionnaire, on demographic and obstetric information, comprised 17 questions that gathered demographic data (age, education level, marital status, etc.) and obstetric information, such as the week of pregnancy, gestational weight gain, condition after cesarean section, the use of assisted reproductive techniques, and contraindications to physical activity during pregnancy. This first part was developed specifically for the present study.

The second part utilized the Polish version of the Get Active Questionnaire for Pregnancy (GAQ-P), developed by the Canadian Society for Exercise Physiology and adapted by A. Szumilewicz et al. [34]. The GAQ-P is an instrument consisting of 20 questions designed to assess contraindications to physical activity during pregnancy as reported by the pregnant woman. Additionally, it allows for the collection of data regarding the woman’s physical activity six months prior to pregnancy, her physical activity during pregnancy, and her plans for physical activity until the end of pregnancy. Furthermore, it includes details about the duration, frequency, level, and type of physical activity. The GAQ-P aimed to evaluate the frequency, intensity, and duration of physical activity during an average week.

### 2.6. Bias

To minimize recall bias and ensure data accuracy, self-reported health information was cross-checked with medical records. This process involved verifying key details, such as diagnoses, treatment history, and medication use, to confirm the consistency and reliability of the information provided by participants. By integrating self-reported data with objective records, the study aimed to enhance the validity of its findings and reduce potential errors associated with participant memory or subjective reporting.

To reduce potential confounding, participants with twin pregnancies were excluded from the study. This decision was based on the understanding that twin pregnancies often present unique health challenges, such as an increased risk of complications like preterm birth, gestational hypertension, and cesarean delivery. By excluding these participants, the study aimed to maintain a more homogeneous sample, ensuring that the results more accurately reflected the experiences and outcomes of individuals with singleton pregnancies.

To further minimize potential confounding factors and improve the accuracy of the findings, patients with placenta previa were also excluded from the study. This condition, characterized by the placenta abnormally covering the cervix, is associated with a higher likelihood of complications such as bleeding during pregnancy, preterm birth, and the necessity for cesarean sections. These complications may significantly alter the course of pregnancy and delivery, potentially impacting physical activity levels. Additionally, the clinical management of placenta previa often involves activity restrictions or prolonged bed rest, which could confound the relationship between pre-pregnancy physical activity, BMI, and the development of gestational diabetes. By excluding these patients, the study ensured a more homogeneous population, allowing for a more accurate evaluation of how pre-pregnancy physical activity and BMI influence the risk of gestational diabetes.

Additionally, the ppBMI (pre-pregnancy body mass index) was calculated based on the data provided by patients in the survey and was verified against medical records to ensure the reliability and accuracy of the collected information. Owing to the standardized prenatal care system in Poland [35], pre-pregnancy weight is recorded in the pregnancy record book (“Karta Przebiegu Ciąży”) early in gestation and subsequently transcribed into electronic medical records. Since early pregnancy weight gain is typically minimal, BMI from the first trimester is often used interchangeably with pre-pregnancy BMI in research when exact preconception measurements are unavailable. This approach helps enhance the accuracy of BMI classification while minimizing recall bias. Subsequently, weight gain during pregnancy was calculated and classified into appropriate categories in accordance with the recommendations of the Institute of Medicine (IOM) [36]. The study excluded participants whose weight gain significantly deviated from normative values. This was a top-down assumption aimed at minimizing the potential impact of extreme values on the study results and improving the consistency of the analyzed study group.

Selection bias is a potential limitation of this study, as it was conducted in a tertiary care center, which may not fully represent the general obstetric population. However, due to regional healthcare restructuring and the closure of lower-tier obstetric facilities, this hospital serves both as a referral center for high-risk pregnancies and as a primary maternity unit for many women with uncomplicated pregnancies. This diverse patient population reduces, but does not completely eliminate, the risk of selection bias. Nevertheless, future multi-center studies would be beneficial to enhance the generalizability of these findings.

The results from the Get Active Questionnaire for Pregnancy (GAQ-P), a validated self-report tool, may be subject to recall bias. This is a common limitation of self-report questionnaires, as participants may misremember the frequency, intensity, or duration of their physical activities, leading to potential over- or under-reporting. Social desirability bias may also play a role, with participants reporting more physical activity than they actually engaged in, in order to conform to perceived societal expectations. To mitigate these biases, future studies should consider incorporating more objective measures, such as accelerometers or heart rate monitoring, to provide more accurate data on physical activity levels.

### 2.7. Variables and Outcome Measures

The primary endpoint of this study was the incidence of gestational diabetes mellitus (GDM). Secondary endpoints included maternal outcomes (pre-pregnancy BMI, gestational weight gain, anemia status, and gestational age at delivery) and neonatal outcomes (birth weight classification and preterm birth rates).

#### 2.7.1. Description of Variables Related to Gestational Diabetes

The independent variable of physical activity during pregnancy was assessed using a self-report questionnaire, specifically based on the Get Active Questionnaire for Pregnancy (GAQ-P) [20]. This questionnaire evaluates the frequency, intensity, and type of physical activity during the pre-pregnancy period and early pregnancy.

Participants were asked to report their engagement in different types of physical activity, which were classified based on the intensity levels [36].

- This tool evaluates the frequency, intensity, and type of physical activity before and during early pregnancy. Participants classified their activity levels based on standardized intensity descriptions and reported their engagement frequency. For clarity, the full questionnaire is included as Supplementary Materials [34].

This detailed assessment helped classify participants into different physical activity levels and evaluate the potential impact of physical activity on gestational diabetes risk [36].

A standardized diagnostic procedure was implemented for all participants in the study. Gestational diabetes mellitus (GDM) was diagnosed using the oral glucose tolerance test (OGTT) in accordance with the diagnostic criteria established by the World Health Organization (WHO) and the International Association of Diabetes and Pregnancy Study Groups (IADPSG). The OGTT was conducted and interpreted based on pregnancy-specific threshold values defined by these guidelines.

In this study, the diagnostic criteria for gestational diabetes mellitus (GDM) were based on the most recent guidelines issued by the World Health Organization (WHO) and the International Association of Diabetes and Pregnancy Study Groups (IADPSG). These internationally recognized guidelines establish pregnancy-specific threshold values for the oral glucose tolerance test (OGTT), which were uniformly applied to all participants.

National guidelines were not utilized due to variations in GDM diagnostic thresholds across different countries. To ensure consistency and comparability of results, the study adhered to the WHO and IADPSG criteria, thereby minimizing potential discrepancies arising from differences in diagnostic standards. The dependent variable of gestational diabetes mellitus (GDM) was diagnosed based on medical records using standard diagnostic criteria (e.g., oral glucose tolerance test, OGTT) and coded as a binary variable (1 = presence of GDM, 0 = absence of GDM) [30]. Gestational diabetes type 1 and type 2 are distinguished based on clinical characteristics, laboratory results, and medical history.

According to the latest guidelines from WHO [37] and IADPSG [37], type 1 diabetes is characterized by autoimmune destruction of pancreatic β-cells, confirmed by the presence of specific autoantibodies (e.g., anti-GAD antibodies). In contrast, type 2 diabetes is primarily associated with insulin resistance and excessive insulin secretion in the early stages of the disease, which can be assessed by measuring fasting insulin and C-peptide levels [37].

In both cases, the diagnosis requires confirmation of impaired glucose tolerance based on OGTT results, using the pregnancy-specific threshold values recommended by WHO and IADPSG [37,38].

#### 2.7.2. Maternal Variables

The first independent variable in the study was pre-pregnancy body mass index (ppBMI), calculated using the following formula: body weight (kg) divided by height squared (m^2^). The ppBMI values were classified according to standard BMI categories based on WHO guidelines and the NIH classification [39,40]:

- Overweight and obesity—BMI greater than or equal to 25.0 and greater than 30 kg/m^2^;
- Normal weight—BMI greater than or equal to 18.5 to 24.9 kg/m^2^;
- Underweight—BMI under 18.5 kg/m^2^;

Pre-pregnancy weight was verified using self-reports and medical records for improved reliability. Gestational weight gain was calculated based on records from the obstetric card by determining the difference between the last recorded weight (from the final prenatal visit or hospital admission) and the weight documented at the first prenatal visit (typically around the 6th week of pregnancy) or the known pre-pregnancy weight. Weight gain during pregnancy (GWG), based on ppBMI, was assessed according to IOM recommendations for optimal maternal and fetal health outcomes. Excessive or insufficient GWG can influence pregnancy risks, including gestational diabetes [36]:

- Underweight (BMI < 18.5 kg/m^2^)—recommended GWG: 12.5–18 kg;
- Normal weight (BMI 18.5–24.9 kg/m^2^)—recommended GWG: 11.5–16 kg;
- Overweight (BMI 25.0–29.9 kg/m^2^)—recommended GWG: 7–11.5 kg;
- Obesity (BMI ≥ 30.0 kg/m^2^)—recommended GWG: 5–9 kg.

According to some researchers, excessive weight gain during pregnancy may be associated with an increased risk of developing gestational diabetes [41,42].

The maternal hemoglobin level in the third trimester was included as an independent variable in the study, as it affects the mother’s health and fetal development.

According to the latest World Health Organization (WHO) guidelines, hemoglobin norms for pregnant women in the third trimester are as follows [43]:

- Normal hemoglobin level: ≥11.0 g/dL (or ≥6.8 mmol/L);
- Mild anemia: 10.0–10.9 g/dL;
- Moderate anemia: 7.0–9.9 g/dL;
- Severe anemia: <7.0 g/dL.

According to some researchers, anemia may be associated with an increased risk of developing gestational diabetes [43,44].

Gestational age was included as an important factor affecting maternal and fetal health. According to the latest guidelines from WHO, the classification of delivery according to gestational age is as follows [45]:

○ On-time birth: 37–42 weeks of gestation;
○ Preterm birth: below 37 weeks of gestation, which can be divided into
  - Extremely preterm: below 28 weeks;
  - Very preterm: from 28 to less than 32 weeks;
  - Moderately preterm: from 32 to 37 weeks.

Preterm births can result in health complications like respiratory distress and developmental delays [40].

#### 2.7.3. Newborn Variables

The infant’s birth weight was included as an outcome variable, as it is an indicator of the newborn’s health and intrauterine development. According to norms, infant birth weight is classified into the following categories [46]:

- Low birth weight (LBW): <2500 g;
- Normal birth weight: 2500–4000 g;
- Fetal macrosomia: >4000 g;
- Extreme macrosomia: >4500 g *.

* In our study, no patients were recorded in this weight category. At the same time, the classification adopted was in accordance with the guidelines of WHO.

Birth weight is an important indicator of both maternal and fetal health. Low birth weight is associated with an increased risk of neonatal health problems, such as respiratory distress, impaired thermoregulation, and hypoglycemia. Conversely, fetal macrosomia may result in complications during delivery, such as birth injuries to the infant and an increased likelihood of cesarean delivery [46].

### 2.8. Statistical Analysis

The results of the data analysis were developed using the statistical software Statistica 13.3. For the entire statistical summary, an alpha level of 0.05 was selected. To address the research questions concerning the relationship between the occurrence of gestational diabetes mellitus and selected variables, logistic regression was utilized. Logistic models were used due to the dichotomous nature of the dependent variable. The diabetes variable, coded as 0—no diabetes and 1—occurrence of diabetes, was considered the dependent variable. Physical activity before pregnancy, pre-pregnancy BMI, gestational weight gain, and the mother’s hemoglobin were used as predictors. In the first attempt to describe diabetes, one-variable models were used. Secondly, multi-variable models were built using all predictors, the strongest predictors in general, and stepwise models, respectively. In logistic regression, the Hosmer–Lemeshow (H–L) test and the determination coefficient (Nagelkerka-R2) were used to assess model fitness and brought up information about percentage of dependent variable explanation. To describe predictor values in models, the Waldt test (z) and odds ratios (Expβ) were utilized. An additional element of the data analysis was the frequency assessment, supported by the chi-square test (χ^2^) and Cramér’s correlation coefficient (V). The chi-square test and Cramer’s coefficient were used to assess the correlation between qualitative variables.

## 3. Results

### 3.1. Characteristics of the Study Group

A total of 205 pregnant women participated in the study, including 42 (20.39%) pregnant women with gestational diabetes and 164 (79.61%) pregnant women without gestational diabetes. Of those with GDM (N = 42), 64.29% (*n* = 27) were pregnant women with type 1 gestational diabetes and 35.71% (*n* = 15) were pregnant women with type 2 gestational diabetes.

The demographic and social data of the women participating in the study are shown in Table 1, which includes details on age, place of residence, education, marital and economic status, and the number of previous births.

#### 3.1.1. Selected Pregnancy-Related Variables

Table 2 shows the standard descriptive values of selected pregnancy-related variables, such as age at delivery, pre-pregnancy BMI, gestational weight gain, newborn birth weight, gestational age at delivery, and maternal hemoglobin level at the time of hospital admission.

#### 3.1.2. Gestational Diabetes Mellitus Prediction

The results of logistic regression analysis are presented to answer the research questions raised about the relationship of selected factors with the incidence of gestational diabetes. Table 3 presents in summary the detailed logistic regression models predicting the incidence of gestational diabetes.

The results showed that the model predicting the occurrence of gestational diabetes, using variables related to the physical activity of women before conception, was well-fitted to the data and explained 4% of the occurrences of gestational diabetes. However, a detailed evaluation of this model indicated that none of the elements determining physical activity (frequency, duration, intensity level) 6 months before conception were significant prognostic factors. Additionally, it was observed that an increase in the frequency and intensity of physical activity was associated with a lower occurrence of gestational diabetes, whereas an increase in the duration of physical activity was linked to a slightly higher occurrence of gestational diabetes. However, it should be noted that the variables related to physical activity did not significantly correlate with gestational diabetes.

The second model, which used categories of pre-pregnancy BMI, was well-fitted to the data and explained 6% of the variability in the occurrence of gestational diabetes. An increase of one category on the pre-pregnancy BMI scale was associated with an 87% increase in the risk of developing gestational diabetes.

The analysis found that pre-pregnancy BMI was a significant predictor of gestational diabetes, with a one-category increase associated with an 87% higher risk. In contrast, physical activity before conception was not a significant predictor, though higher frequency and intensity were linked to a lower risk, while longer duration showed a slight increase in occurrence. However, these associations were not statistically significant. The models were well-fitted to the data, explaining 4% (physical activity) and 6% (pre-pregnancy BMI) of the variability in gestational diabetes occurrence.

In an attempt to assess the prediction of gestational diabetes, general models were also constructed using all the previously presented predictive factors. Table 4 shows logistic regression models predicting postpartum diabetes in the general model using the enter method and the general model using the stepwise method.

The general model using all the predictive factors was well-fitted to the data and explained 17% of the occurrences of gestational diabetes. In the model, significant predictive factors were pre-pregnancy BMI (ppBMI) and weight gain during pregnancy. The second model, built using the stepwise method, only utilized the strongest predictive factors. This model was also well-fitted to the data and explained 14% of the variability in gestational diabetes. An increase of one category on the ppBMI scale in the model was associated with a 163% increase in the odds of developing gestational diabetes. Conversely, as weight gain during pregnancy increased by one unit on the interpretation scale, the risk of developing gestational diabetes decreased.

The described variables, such as pre-pregnancy BMI and weight gain during pregnancy, were also subjected to a detailed analysis of their frequency in the occurrence of postpartum diabetes. A summary of this analysis is presented in Table 5.

Among women with underweight pre-pregnancy BMI, only 2.38% developed gestational diabetes, compared to 42.86% in the group with normal pre-pregnancy BMI and 54.76% in women who were overweight.

These results suggest that being overweight before pregnancy is associated with a significantly higher risk of developing gestational diabetes, affecting almost 55% of women, while the risk in women who are underweight is much lower. The group with normal pre-pregnancy weight has a relatively lower incidence of gestational diabetes (29.88%), indicating that pre-pregnancy BMI plays a crucial role in the development of gestational diabetes.

#### 3.1.3. Gestational Diabetes Mellitus Complications

The second area of data analysis was an attempt to assess the correlates of physical activity of women before pregnancy that are also complications associated with the occurrence of gestational diabetes. The first factor evaluated was the birth weight of the child. Table 6 presents a summary of the results obtained in this assessment.

The analysis showed that the relationship between birth weight and the frequency (χ^2^(6) = 2.93, *p* = 0.817, V = 0.08), intensity (χ^2^(4) = 5.26, *p* = 0.261, V = 0.11), and duration (χ^2^(6) = 4.18, *p* = 0.652, V = 0.11) of physical activity before pregnancy was not statistically significant. A similar assessment of the level of physical activity before pregnancy was conducted in relation to the mode of delivery, the summary of which is presented in Table 7.

Here, too, the analysis showed no significant relationship between the mode of delivery and the frequency (χ^2^(3) = 2.26, *p* = 0.521, V = 0.11), intensity (χ^2^(2) = 5.09, *p* = 0.078, V = 0.16), or duration (χ^2^(3) = 1.68, *p* = 0.640, V = 0.09) of physical activity before pregnancy. The final area of analysis regarding the level of physical activity before pregnancy was preterm birth. Table 8 presents a summary of the results of this assessment.

The relationship between the frequency (χ^2^(3) = 3.28, *p* = 0.350, V = 0.13), intensity (χ^2^(2) = 4.59, *p* = 0.100, V = 0.15), and duration (χ^2^(3) = 2.39, *p* = 0.450, V = 0.11) of physical activity before pregnancy was found to be statistically insignificant in relation to preterm birth.

## 4. Discussion

Our study provides significant insights into the relationship between pre-pregnancy body mass index (ppBMI), preconception physical activity levels, and the risk of gestational diabetes mellitus (GDM). First, we demonstrated that a higher ppBMI significantly increases the risk of GDM, with an increase of one ppBMI category being associated with an 87% higher risk. This finding aligns with previous studies highlighting the detrimental effects of obesity on glucose metabolism in pregnant women [21]. Second, while preconception physical activity was not identified as a significant predictor of GDM, higher exercise frequency and intensity were associated with a reduced risk. However, paradoxically, a longer duration of physical activity correlated with a higher risk, which was an unexpected observation. Third, regression model analysis indicated that the best-fitting models, incorporating ppBMI and gestational weight gain, explained 17% and 14% of the variance in GDM incidence, respectively, underscoring the relevance of these factors for public health and prevention strategies [47].

This study did not identify preconception physical activity as a statistically significant predictor of gestational diabetes mellitus (GDM). However, it is important to acknowledge the extensive body of research demonstrating that regular physical activity enhances insulin sensitivity and contributes to overall maternal health during pregnancy [15,26,48]. Numerous studies have shown that engaging in moderate-intensity exercise before and during pregnancy can improve glucose metabolism, reduce insulin resistance, and lower the risk of complications associated with GDM [21,26,48,49]. While our findings did not establish a direct association, this may be due to study limitations such as sample size, self-reported physical activity data, or unmeasured lifestyle factors.

It was unexpected that a longer duration of physical activity before pregnancy would be associated with an increased risk of GDM. One possible explanation is that intense exercise without adequate energy balance may have contributed to metabolic disturbances and heightened oxidative stress [26,48,49,50,51]. Some studies have reported similar findings, where excessive physical activity was linked to increased insulin resistance [49,52,53,54]. A potential confounding factor in the relationship between pre-pregnancy physical activity and gestational diabetes mellitus (GDM) risk is the presence of underlying metabolic conditions. More intense pre-pregnancy exercise is often associated with overall better metabolic health; however, in some cases, women who engage in high levels of physical activity may do so as a response to pre-existing metabolic disturbances, such as polycystic ovary syndrome (PCOS), insulin resistance, or obesity. These conditions are independently linked to an increased risk of GDM, potentially complicating the observed relationship between physical activity and GDM risk [55]. Furthermore, individuals with metabolic disorders may experience altered hormonal and inflammatory profiles that influence glucose metabolism, making them more susceptible to GDM regardless of their physical activity levels. Additionally, variations in body composition, such as differences in lean muscle mass and fat distribution, could further mediate the relationship between exercise and GDM risk [56]. Another consideration is that women with a history of metabolic dysfunction might receive medical advice to increase physical activity before pregnancy, leading to a form of reverse causation in observational studies. To better understand these potential confounding effects, future research should incorporate more comprehensive assessments of pre-pregnancy metabolic health, including biomarkers of insulin sensitivity, lipid profiles, and inflammatory markers. Adjusting for these factors in statistical models may help clarify whether the observed associations between pre-pregnancy exercise and GDM risk are independent or primarily driven by underlying metabolic conditions [55,56]. Although dietary factors were not measured in this study, it is possible that unmeasured dietary variables could act as confounders in the observed associations between physical activity and gestational diabetes risk. Diet is known to play a significant role in metabolic health, and it may interact with physical activity in influencing GDM risk [15].

Our results also reveal significant differences in GDM risk between women with varying ppBMI categories. While underweight women exhibited the lowest risk of GDM (2.38%), overweight and obese women demonstrated risks of 42.86% and 54.76%, respectively. These findings corroborate previous studies highlighting the substantial impact of body weight on glucose metabolism during pregnancy [26].

Future research should consider stratifying analyses by BMI category to determine whether the effects of physical activity on GDM risk vary depending on weight status. This approach could help clarify whether the associations observed in our study differ among normal-weight, overweight, and obese individuals. While our study primarily aimed to assess the overall relationship between physical activity and GDM, future investigations focusing on BMI subgroups could provide more targeted insights and inform personalized prevention strategies. The results of our study did not confirm an association between low hemoglobin levels in the third trimester and the occurrence of GDM, a finding that contrasts with the study by Wang et al., which suggests a role for anemia in the pathogenesis of this condition [57]. Other studies have proposed potential mechanisms, including increased oxidative stress and insulin resistance related to iron deficiency, but our study did not demonstrate these associations. This discrepancy may be attributed to the fact that we assessed anemia at the end of pregnancy, as we aimed to ensure consistent and uniform data for analysis across all participants. In contrast, data from the early stages of pregnancy medical records were derived from different laboratories and collected at various time points, which would not allow for a reliable comparison of results. Moreover, our study found no significant correlations between pre-pregnancy physical activity and the mode of pregnancy completion or the risk of preterm delivery, which may suggest that other factors, such as maternal health status, play a more substantial role. These findings contrast with previous research indicating that regular physical activity can reduce the risk of cesarean section and preterm labor [58]. Possible differences may be attributed to variations in research methodology, demographic characteristics, and differing levels of physical activity within the study population.

The analysis revealed that the relationship between birth weight and the frequency, intensity, and duration of physical activity before pregnancy was not statistically significant. This result can be interpreted as positive, suggesting that well-controlled gestational diabetes does not have an adverse impact on birth weight. Although our original hypothesis aimed to investigate whether excessive weight gain during pregnancy could lead to the development of gestational diabetes, our findings indicate that women with more controlled weight gain during pregnancy were more frequently included in the group with gestational diabetes. This may suggest that, despite the diagnosis of gestational diabetes, our patients were under careful management by endocrinologists, obstetricians, and dietitians. This comprehensive care likely prevented excessive weight gain during pregnancy, reducing the risk of preterm delivery and the birth of infants with low birth weight. Furthermore, the study did not show an increased risk of cesarean section in our sample, a positive outcome reflecting the effective control of diabetes and early intervention for this group of patients, which in turn mitigated the development of complications. This highlights the importance of multidisciplinary care in minimizing adverse outcomes in pregnancies complicated by gestational diabetes.

While we were unable to consider additional factors such as family history of diabetes, diet, or insulin sensitivity in the current study, we acknowledge their potential influence on GDM risk. These variables should be incorporated into future research to enhance our understanding of the complex interplay of factors contributing to gestational diabetes.

Future studies should explore the impact of post-pregnancy lifestyle changes, including diet and physical activity, on the recurrence of gestational diabetes mellitus (GDM), as such interventions may play a critical role in preventing subsequent GDM episodes and improving long-term maternal health.

### 4.1. Strengths of the Study

A key strength of this study is the inclusion of both retrospective and prospective data collection methods, which allowed for a comprehensive evaluation of the relationship between pre-pregnancy BMI, physical activity, and gestational diabetes mellitus (GDM). The use of objective medical records to assess pre-pregnancy BMI and GDM diagnosis minimized recall bias, ensuring the reliability of the data. Additionally, the inclusion of the validated Polish version of the Get Active Questionnaire for Pregnancy (GAQ-P) provided a standardized assessment of physical activity levels, improving the consistency and comparability of self-reported data.

Another strength of this study is the relatively homogenous study population, as women with multiple pregnancies were excluded to reduce potential confounding effects on pregnancy outcomes. This approach allowed for a more precise evaluation of the associations between lifestyle factors and GDM risk. Furthermore, the use of multiple statistical methods, including logistic regression and Cramér’s V coefficient, ensured a robust analysis of the data. These methodological strengths enhance the validity of the findings and provide a strong foundation for future research on modifiable risk factors for GDM.

### 4.2. Limitation

Several limitations should be noted. First, physical activity data were based on self-reporting, which may introduce reporting biases. Additionally, we did not account for dietary factors, which are important modifiers of GDM risk. The lack of significant associations between physical activity and preterm delivery or mode of pregnancy completion could be due to insufficient statistical power.

While the Get Active Questionnaire for Pregnancy (GAQ-P) is a validated tool, self-reported physical activity data inherently rely on participants’ memory and subjective perception, which may introduce inaccuracies. One of the key challenges is the tendency for over- or underestimation of both exercise duration and intensity due to recall bias. Participants may misinterpret the intensity of their activity, as perceived exertion varies among individuals. Additionally, differences in understanding or defining moderate versus vigorous activity could lead to inconsistencies in reporting. Estimating total time spent exercising can also be imprecise, as individuals might round up or down their activity durations based on general habits rather than exact measurements.

Furthermore, future studies could benefit from using more precise and objective tools, such as the Pregnancy Physical Activity Questionnaire or accelerometers, to assess physical activity levels with greater accuracy. These tools can provide a more reliable assessment of the frequency, intensity, and duration of physical activity compared to self-reported data. Additionally, standardized fitness tests and heart rate monitoring could be employed to determine the intensity of physical activity more objectively. Another limitation of this study is the lack of differentiation between structured exercise and incidental movement, as the Get Active Questionnaire for Pregnancy (GAQ-P) captures overall physical activity without distinguishing between these types of activity. This could introduce variability in the data, as structured exercise and incidental movement may have differing effects on maternal health and gestational diabetes risk.

Another limitation of this study is its single-center design, which may affect the generalizability of the findings. However, due to the declining birth rate in Poland, many lower-tier obstetric facilities in the study region have been closed, making multi-center studies increasingly challenging. As a result, the study hospital serves as both a specialized referral center and a primary maternity unit for a diverse patient population, including women with both high-risk and uncomplicated pregnancies. While this setting allowed for a well-defined study population, future multi-center studies are needed to confirm and expand upon these findings in different clinical settings.

## 5. Conclusions

The results of this study highlight the role of pre-pregnancy body mass index (BMI) as a significant risk factor for the development of gestational diabetes, emphasizing the need for targeted interventions focused on weight management before conception. The analysis showed that women with a higher pre-pregnancy BMI are significantly more likely to develop GDM, underscoring the importance of preconception counseling and lifestyle modifications to achieve and maintain a healthy weight.

While physical activity is widely recognized for its health benefits, including improving metabolic function and reducing the risk of chronic diseases, our study suggests that its direct impact on gestational diabetes mellitus (GDM) risk may be more limited in this context. The lack of a significant association between pre-pregnancy physical activity and GDM incidence in our study indicates that while regular exercise remains important for overall health, weight management through maintaining a healthy BMI may play a more crucial role in preventing GDM. However, this does not diminish the valuable role of physical activity in overall health promotion, and future research may further explore its contribution to GDM risk. These findings suggest the paradox that a balanced, comprehensive approach to GDM prevention, where both weight management and physical activity are emphasized, may be most effective. Our study did not find significant associations between pre-pregnancy physical activity and pregnancy outcomes such as birth weight, mode of delivery, or preterm birth. These findings contrast with previous research, which reported that women engaging in regular physical activity before pregnancy had a lower risk of very early preterm birth and a higher likelihood of vaginal delivery [58]. The discrepancies between our results and prior studies may be attributed to several factors. First, our study relied on self-reported physical activity data, which is subject to recall bias and variability in individual perception of exercise intensity. Second, we did not account for dietary factors, which are known to play a crucial role in pregnancy outcomes and may interact with physical activity levels. Third, differences in study populations, sample sizes, and methodological approaches could also contribute to the observed inconsistencies. Given these findings, future research should employ objective measures of physical activity and consider additional lifestyle factors, including nutrition and metabolic health, to better elucidate the complex interplay between physical activity and pregnancy outcomes. Considering fetal weight, mode of delivery, and weight gain during pregnancy, our findings may also be attributed to the fact that well-managed and controlled gestational diabetes does not necessarily impact pregnancy and neonatal outcomes. This is a positive observation, as it suggests that effective management strategies can mitigate potential adverse effects, reinforcing the importance of early diagnosis, medical supervision, and lifestyle interventions in optimizing maternal and neonatal health. Therefore, public health strategies should focus on comprehensive preconception care, including nutritional counseling, weight management programs, and early screening for women at higher risk. Therefore, public health strategies should focus on comprehensive preconception care, including nutritional counseling, weight management programs, and early screening for women at higher risk. In light of the growing global obesity epidemic, these interventions could have significant benefits not only for reducing the risk of gestational diabetes mellitus (GDM) but also for improving overall maternal and child health. Obesity, which is closely linked to a higher risk of GDM, remains a major public health challenge worldwide, with increasing rates of overweight status and obesity among women of reproductive age. Promoting healthy weight before pregnancy and supporting women in achieving and maintaining a healthy body mass index (BMI) could lead to better pregnancy outcomes and lower healthcare costs associated with pregnancy complications, such as GDM, preterm birth, and cesarean delivery.

In addition to addressing obesity, the promotion of physical activity, especially regular exercise before and during pregnancy, could help mitigate some of the risks associated with GDM and improve maternal health. Public health campaigns that emphasize the benefits of physical activity, healthy eating, and early screening may reduce the incidence of GDM and related complications, leading to healthier pregnancies and better long-term health for both mothers and children. Future research should further explore the complex relationships between lifestyle factors, metabolic health, and genetic predispositions to develop more effective preventive strategies. A better understanding of these relationships may help optimize maternal health and improve pregnancy outcomes, ultimately reducing the burden of gestational diabetes for both women and healthcare systems.

## Figures and Tables

**Table 1:** Characteristics of the study group.

Variable	Value	N	%
	<25 years	20	9.39
Age	25–30 years	87	40.85
	>30 years	106	49.76
	Village	34	16.50
	City up to 50,000 inhabitants	30	14.56
	City of 50,000 to 100,000 inhabitants	23	11.17
Place of residence	City of 100,000 to 250,000 inhabitants	7	3.40
	City of 100,000 to 500,000 inhabitants	6	2.91
	City over 500,000 inhabitants	104	50.49
	Primary	5	2.43
	Basic vocational	14	6.80
Education	Secondary	44	21. 36
	Higher	143	69.42
	Miss	47	22.82
	Married	157	76.21
Marital status	Widow	1	0.49
	Divorced	1	0.49
Economic status	Average	36	17.48
	Good	169	82.04
	One child	101	49.03
	Two children	52	25.24
Number of children	Three children	27	13.11
	Four or more children	26	12.62

**Table 2:** Standard descriptive values of chosen pregnancy-related variables.

Tested Variable	M ± SD
Age at delivery	31.15 ± 4.92
Pre-pregnancy BMI	24.48 ± 5.29
Gestational weight gain	13.42 ± 5.62
Birth weight	3332.82 ± 602.08
Gestational age at birth (weeks)	38.80 ± 2.78
Maternal hemoglobin	12.47 ± 1.40

**Table 3:** Detailed logistic regression models in predicting gestational diabetes mellitus.

Model	Predictor		B	SE	z	Expβ	*p*
1	Physical activity 6 months before the pregnancy	Frequency	−0.09	0.28	0.11	0.91	0.746
		Intensity	−0.88	0.47	3.46	0.42	0.063
		Duration	0.17	0.28	0.37	1.18	0.543
2	Pre-pregnancy BMI		0.62	0.21	8.70	1.87	0.003
3	Gestational weight gain		−0.36	0.21	2.81	0.70	0.093
4	Mother hemoglobin		0.01	0.12	0.01	1.01	0.935

Model 1: H-L = 4.49; *p* = 0.482. Nagelkerka-R^2^ = 0.04. Model 2: H-L = 0.36; *p* = 0.547. Nagelkerka-R^2^ = 0.06. Model 3: H-L = 0.01; *p* = 0.923. Nagelkerka-R^2^ = 0.02. Model 4: H-L = 1.60; *p* = 0.979. Nagelkerka-R^2^ = 0.00. B—non-standardized coefficient, SE—standard error, z—Wald statistic, Expβ—odds ratio, *p*—significance.

**Table 4:** General logistic regression models in predicting postpartum diabetes.

Model	Predictor		B	SE	z	Expβ	*p*
1	Physical activity before pregnancy	Frequency	−0.13	0.29	0.21	0.88	0.650
		Intensity	−0.89	0.49	3.26	0.41	0.071
		Duration	0.27	0.28	0.92	1.31	0.338
	Pre-pregnancy BMI		0.93	0.26	12.33	2.52	<0.001
	Pregnancy weigh gain		−0.78	0.26	9.36	0.46	0.002
	Maternal hemoglobin		0.02	0.12	0.02	1.02	0.878
2	Pre-pregnancy BMI		0.97	0.26	14.13	2.63	<0.001
	Gestational weight gain		−0.75	0.25	8.90	0.47	0.003

Model 1: H-L = 6.37; *p* = 0.606, Nagelkerka-R^2^ = 0.17. Model 2: H-L = 5,19; *p* = 0.268, Nagelkerka-R^2^ = 0.14. B—non-standardized coefficient, SE—standard error, z—Wald statistic, Expβ—odds ratio, *p*—significance.

**Table 5:** Frequency analysis of gestational diabetes considering ppBMI and gestational weight gain.

Health Condition	Pre-Pregnancy BMI					
	Underweight		Normal Weight		Overweight	
	*n*	%	n	%	n	%
No GDM	6	3.66	109	66.46	49	29.88
GDM	1	2.38	18	42.86	23	54.76
Health condition	Gestational weigh gain					
	Too low		Accurate		Too large	
	*n*	%	n	%	n	%
No GDM	46	28.05	52	31.71	66	40.24
GDM	17	40.48	13	30.95	12	28.57

**Table 6:** Frequency analysis of physical activity before pregnancy considering birth weight.

Physical Activity Before Pregnancy		Birth Weight to Gestational Age					
		Too Small		Appropriate		Too Large	
		*n*	%	*n*	%	*n*	%
Frequency	0	3	7.89	30	78.95	5	13.16
	1–2	6	7.23	70	84.34	7	8.43
	3–4	5	7.25	60	86.96	4	5.80
	5–7	2	13.33	11	73.33	2	13.33
Intensity	Low	4	5.80	55	79.71	10	14.49
	Moderate	10	8.06	105	84.68	9	7.26
	High	2	15.38	11	84.62	0	0.00
Duration	<20	7	12.07	44	75.86	7	12.07
	20–30	1	3.45	26	89.66	2	6.90
	30–60	5	5.95	71	84.52	8	9.52
	>60	3	8.57	30	85.71	2	5.71

**Table 7:** Frequency analysis of the physical activity before pregnancy considering mode of birth.

Physical Activity Before Pregnancy		Mode of Birth			
		Vaginal Birth		C-Section	
		*n*	%	*n*	%
Frequency	0	2	5.56	34	94.44
	1–2	12	14.81	69	85.19
	3–4	9	13.43	58	86.57
	5–7	1	8.33	11	91.67
Intensity	Low	4	6.06	62	93.94
	Moderate	17	14.17	103	85.83
	High	3	27.27	8	72.73
Duration	<20	6	10.91	49	89.09
	20–30	2	7.69	24	92.31
	30–60	10	12.05	73	87.95
	>60	6	18.18	27	81.82

**Table 8:** Frequency analysis of the physical activity before pregnancy considering pregnancy termination due date.

Physical Activity Before Pregnancy		Pregnancy Termination Due Date			
		Premature		On-Time	
		*n*	%	*n*	%
Frequency	0	23	60.53	15	39.47
	1–2	53	63.86	30	36.14
	3–4	52	75.36	17	24.64
	5–7	10	66.67	5	33.33
Intensity	Low	48	69.57	21	30.43
	Moderate	79	63.71	45	36.29
	High	12	92.31	1	7.69
Duration	<20	35	60.34	23	39.66
	20–30	22	75.86	7	24.14
	30–60	58	69.05	26	30.95
	>60	24	68.57	11	31.43

## Data Availability

The original contributions presented in the study are included in the article; further inquiries can be directed to the corresponding author.

## Supplementary Materials

The following supporting information can be downloaded at: https://www.mdpi.com/article/10.3390/nu17050786/s1. 1. STROBE checklist; 2. Get Active Questionnaire for Pregnancy (GAQ-P) Polish version.

## Abbreviations

The following abbreviations are used in this manuscript:

GDM	gestational diabetes
GDM2	gestational diabetes type 2
GAQ-P	Get Active Questionnaire for Pregnancy
BMI	body mass index
ppBMI	pre-pregnancy body mass index
WMA	World Medical Association
GUS	(pol. Główny Urząd Statystyczny, Central Statistical Office)
IOM	Institute of Medicine
OGTT	oral glucose tolerance test
WHO	World Health Organization
IADPSG	International Association of Diabetes and Pregnancy Study Groups
NIH	National Institutes of Health
GWG	gestational weight gain
Hb	hemoglobin
LBW	low birth weight
