# Supplementary material for: Prenatal Physical Activity, Pre-Pregnancy BMI, and Their Relationship with Gestational Diabetes: A Retrospective-Prospective Single-Center Study
Source: Nutrients. 2025 Feb 24;17(5):786. doi: 10.3390/nu17050786 (PMC11901483; doi:10.3390/nu17050786)
Supplement: Supplementary file 1 [file nutrients-17-00786-s001.zip › GAQ-P-Polish.pdf]

KWESTIONARIUSZ BĄDŹ AKTYWNA W CIĄŻY

CSEP | SCPE

IMIĘ I NAZWISKO (+ IMIĘ I NAZWISKO RODZICA/OPIEKUNA PRAWNEGO, JEŚLI DOTYCZY) [PROSZĘ WYPEŁNIĆ DRUKOWANYMI LITERAMI]:			
DZISIEJSZA DATA (DD/MM/RRRR):	TERMIN PORODU (DD/MM/RRRR):	TYDZIEŃ CIĄŻY:	WIEK:

Aktywność fizyczna w czasie ciąży ma wiele korzyści zdrowotnych i najczęściej nie wiąże się z ryzykiem ani dla Pani, ani dla Pani dziecka. Jednak w niektórych sytuacjach aktywność fizyczna nie jest zalecana. Ten kwestionariusz ma pomóc Pani w podjęciu decyzji, czy przed rozpoczęciem lub kontynuacją aktywności fizycznej powinna Pani porozmawiać z lekarzem lub położną prowadzącymi ciążę.

Proszę odpowiedzieć TAK lub NIE na każde pytanie, na tyle, na ile Pani potrafi. **Jeśli Pani stan zdrowia zmieni się wraz z przebiegiem ciąży, powinna Pani ponownie wypełnić ten kwestionariusz.**

		Tak	Nie
1.	Czy w aktualnej ciąży wystąpiły u Pani:		
	a. Łagodne, umiarkowane lub ciężkie choroby układu oddechowego lub sercowo-naczyniowego (np. przewlekłe zapalenie oskrzeli)?		
	b. Padaczka, która nie jest ustabilizowana?	T	N
	c. Cukrzyca typu 1, która nie jest ustabilizowana lub poziom cukru we krwi jest poza zalecanym zakresem?	T	N
	d. Choroba tarczycy, która nie jest ustabilizowana lub hormony tarczycy określające czynność tarczycy są poza zalecanym zakresem?	T	N
	e. Zaburzenia odżywiania lub niedożywienie?	T	N
	f. Bliźnięta (28 tydzień ciąży lub później)? Lub ciąża trojacza albo liczniejsza ciąża mnoga?	T	N
	g. Niski poziom czerwonych krwinek (niedokrwistość) z objawami zmęczenia i/lub zawrotami głowy?	T	N
	h. Wysokie ciśnienie tętnicze krwi (stan przedrzucawkowy, nadciśnienie ciążowe lub przewlekłe nadciśnienie, które nie jest ustabilizowane)?	T	N
	i. Dziecko, które rośnie zbyt wolno (wewnątrzmaciczne ograniczenie wzrastania płodu)?	T	N
	j. Niewyjaśnione krwawienie z dróg rodnych, pęknięcie błon płodowych lub poród rozpoczęty przed 37 tygodniem ciąży?	T	N
	k. Łożysko, które częściowo lub całkowicie zakrywa szyjkę macicy (łożysko przodujące)?	T	N
	l. Niewydolna szyjka macicy?	T	N
	m. Szew lub taśma wzmacniająca szyjkę macicy?	T	N
2.	Czy w poprzednich ciążach wystąpiły u Pani:		
	a. Nawracające poronienia (utrata dziecka przed 20 tygodniem ciąży dwa lub więcej razy z rzędu)?	T	N
	b. Przedwczesny poród (przed 37 tygodniem ciąży)?	T	N
3.	Czy ma Pani inne problemy zdrowotne, które mogą wpływać na Pani zdolność do aktywności fizycznej podczas ciąży? Proszę podać co to za problemy zdrowotne:	T	N
4.	Czy jest jakiś inny powód, dla którego obawia się Pani aktywności fizycznej w czasie ciąży?		

Przejdź do strony nr 2 Proszę opisać Pani poziom aktywności fizycznej

© Canadian Society for Exercise Physiology (CSEP)

Proszę Opisać Pani Poziom Aktywności Fizycznej

CSEP | SCPE

W czasie typowego tygodnia, w jakich rodzajach aktywności fizycznej Pani uczestniczy (np. pływanie, spacery, trening oporowy/ ćwiczenia kształtujące poszczególne części ciała, joga)?

____

____

W odniesieniu do tego samego, typowego tygodnia proszę opisać, jak często i jak długo PRZECIĘTNIE uprawia Pani aktywność fizyczną o niskiej, umiarkowanej lub wysokiej intensywności. Proszę zobaczyć definicje intensywności w ramce poniżej.

PRZECIĘTNIE:	CZĘSTOŚĆ (ILE RAZY W TYGODNIU)	INTENSYWNOŚĆ (PROSZĘ PRZECZYTAĆ DEFINICJE PONIŻEJ)	CZAS TRWANIA (ILE MINUT NA SESJĘ ĆWICZEŃ)
Na ile była Pani aktywna fizycznie w **ciągu sześciu miesięcy przed ciążą?**	☐ 0 ☐ 3-4 ☐ 1-2 ☐ 5-7	☐ niska ☐ umiarkowana ☐ wysoka	☐ <20 ☐ 31-60 ☐ 20-30 ☐ >60
Na ile była Pani aktywna fizycznie do tej pory **podczas tej ciąży**?	☐ 0 ☐ 3-4 ☐ 1-2 ☐ 5-7	☐ niska ☐ umiarkowana ☐ wysoka	☐ <20 ☐ 31-60 ☐ 20-30 ☐ >60
Jakie są Pani cele związane z aktywnością fizyczną **do końca ciąży**?	☐ 0 ☐ 3-4 ☐ 1-2 ☐ 5-7	☐ niska ☐ umiarkowana ☐ wysoka	☐ <20 ☐ 31-60 ☐ 20-30 ☐ >60

Aktywność fizyczna o niskiej intensywności: Porusza się Pani, ale bez pocenia się i ciężkiego oddychania, np. chodzenie do sklepu lub lekkie prace w ogrodzie.

Aktywność fizyczna o umiarkowanej intensywności: Pani tętno wzrasta i może Pani się pocić lub ciężko oddychać. Może Pani mówić, ale nie może śpiewać, np. szybki marsz.

Aktywność fizyczna o wysokiej intensywności: Pani tętno znacznie wzrasta, czuje się Pani zgrzana i spocona, nie może Pani powiedzieć więcej niż kilka słów bez robienia przerw na oddech, np. szybka jazda na rowerze stacjonarnym lub bieganie.

Ogólne Zalecenia Dotyczące Aktywności Fizycznej w Czasie Ciąży

Należy postępować zgodnie z zaleceniami dotyczącymi aktywności fizycznej w czasie ciąży zawartymi w polskich wytycznych PTGiP z 2023 (**https://journals.viamedica.pl/ginekologia_polska/article/view/95954**) i w kanadyjskich wytycznych z 2019 r. (**csepguidelines.ca/pregnancy**)

Kobietom w ciąży zaleca się tygodniowo co najmniej 150 minut aktywności fizycznej o umiarkowanej intensywności (np. trening oporowy/ ćwiczenia kształtujące poszczególne części ciała, szybki marsz, pływanie, prace w ogrodzie), rozłożonej na trzy lub więcej dni w tygodniu. **Jeśli planuje Pani wziąć udział w intensywnej aktywności fizycznej lub być aktywną fizycznie na wysokości powyżej 2500 m n.p.m., należy skonsultować się ze swoim lekarzem lub położną prowadzącymi ciążę**. Jeśli ma Pani jakiekolwiek pytania dotyczące aktywności fizycznej podczas ciąży, należy skonsultować się z wykwalifikowanym instruktorem lub trenerem ćwiczeń w ciąży lub lekarzem czy położną prowadzącymi ciążę. Może to pomóc upewnić się, że Pani aktywność fizyczna jest bezpieczna i odpowiednia dla Pani.

Oświadczenie

Zgodnie z moją najlepszą wiedzą, wszystkie informacje podane przeze mnie w tym kwestionariuszu są prawdziwe. **Jeśli mój stan zdrowia ulegnie zmianie, wypełnię ten kwestionariusz ponownie.**

☐ **Odpowiedziałam NIE na wszystkie pytania na stronie 1. Proszę umieścić datę i podpisać poniższe oświadczenie. Aktywność fizyczna jest zalecana.**

☐ **Odpowiedziałam TAK na jedno lub więcej pytań na stronie 1 i porozmawiam z moim lekarzem lub moją położną prowadzącymi ciążę przed rozpoczęciem lub kontynuowaniem aktywności fizycznej**. Aby rozpocząć rozmowę z lekarzem lub położną, można skorzystać z „Formularza konsultacji z lekarzem lub położną w zakresie aktywności fizycznej w ciąży" (csepguidelines.ca/pregnancy).

☐ **Rozmawiałam z moim lekarzem lub położną prowadzącymi ciążę, który zalecili mi aktywność fizyczną podczas ciąży.** Proszę umieścić datę i podpisać poniższe oświadczenie.

IMIĘ I NAZWISKO (IMIĘ I NAZWISKO RODZICA/OPIEKUNA PRAWNEGO JEŚLI DOTYCZY) (PROSZĘ WYPEŁNIĆ DRUKOWANYMI LITERAMI)		PODPIS (LUB PODPIS RODZICA LUB OPIEKUNA PRAWNEGO, JEŚLI DOTYCZY):
DZISIEJSZA DATA (DD/MM/RRRR):	NUMER TELEFONU (OPCJONALNIE)	EMAIL (OPCJONALNIE)

© Canadian Society for Exercise Physiology (CSEP)
